# Os Pacientes com Síndrome de Heyde Deveriam Sofrer Intervenção Valvar Precoce?

**DOI:** 10.36660/abc.20210734

**Published:** 2021-09-01

**Affiliations:** 1 Universidade Federal da Paraíba João Pessoa PB Brasil Universidade Federal da Paraíba, João Pessoa, PB – Brasil; 2 Faculdade Ciências Médicas de Minas Gerais Belo Horizonte MG Brasil Faculdade Ciências Médicas de Minas Gerais, Belo Horizonte, MG – Brasil; 3 Universidade de São Paulo Hospital das Clínicas da Faculdade de Medicina Instituto do Coração (InCor) São Paulo Brasil Instituto do Coração (InCor) do Hospital das Clínicas da Faculdade de Medicina da Universidade de São Paulo, São Paulo – Brasil

**Keywords:** Estenose da Valva Aórtica, Hemorragia Gastrointestinal/complicações, Intervenção Coronária Percutânea, Comorbidades, Mortalidade

O perfil epidemiológico do paciente com estenose aórtica vem mudando com o envelhecimento da população, caracterizando-se por aumento das comorbidades associadas.^1^ Graças à revolução do tratamento valvar aórtico percutâneo, muitos pacientes que antes teriam uma sobrevida e uma qualidade de vida limitada, vêm se beneficiando da possibilidade de um tratamento curativo. Por outro lado, os desafios clínicos que surgem nessa população reforçam não somente a relevância consolidada do *heart team*, mas também de outras especialidades como oncologia e hematologia.

O interessante estudo publicado na atual edição reforça a importância da associação entre a síndrome de Heyde e estenose aórtica, com aumento de sangramento nessa população. Porém é importante realçar nesse contexto as comorbidades frequentemente associadas e que podem contribuir para eventuais hemorragias digestivas, tais como a associação entre estenose aórtica com neoplasia, pois ambas compartilham os mesmos fatores de risco, em particular, a neoplasia de cólon.^2^ Esta apresenta-se com os sinais clínicos cardinais: anemia e a hemorragia digestiva baixa. Nesse contexto, é mandatória a exclusão de neoplasia, mesmo na suspeita de Síndrome de Heyde. Ainda podemos descrever outras comorbidades frequentemente associadas^3^ que agregam risco de sangramento pela terapêutica utilizada, como a doença arterial coronária que, habitualmente, utiliza a antiagregação plaquetária, ou a dupla antiagregação após a realização de intervenção coronariana percutânea com stent eluído em droga. Outro exemplo é a prevalência aumentada de fibrilação atrial.^4^ Nesse cenário, quando a Síndrome de Heyde está presente, ocorre uma maximização intrínseca de sangramento uma vez que se utiliza a anticoagulação. Outra condição clínica que vem crescendo na população idosa é a amiloidose cardíaca, principalmente por transtiretina. A associação com a estenose aórtica grave é mais comum do que se imaginava, variando entre 12 e 16%.^5^^,^^6^ Assim como a estenose aórtica, a sua prevalência é maior na população idosa; a fibra amiloide atua diretamente na cascata da coagulação, favorecendo o surgimento de eventos hemorrágicos espontâneos.^7^ Por último, não relacionado com aumento de sangramento, mas com o impacto que isso possa ocasionar, a fragilidade do paciente com estenose aórtica.^8^ Um paciente com estenose aórtica grave e considerado frágil, tem uma menor reserva orgânica para suportar um eventual sangramento grave. Sabe-se que a necessidade de transfusão sanguínea impacta negativamente na morbidade e mortalidade dessa população.^9^

O trabalho de Rosa et al.^10^ reforça a importância de uma associação negligenciada e com uma elevada taxa de sangramento grave e necessidade de transfusão na admissão, com risco elevado de mortalidade. A recomendação atual para realização de rastreio com colonoscopia de câncer colorretal reduziu para 45 anos segundo a *U.S. Preventive Services Task Force*. Além disso, no nosso meio, o acesso a procedimentos endoscópicos não é massivamente disponível para grande parte da população o que agrega um risco aumentado, uma vez que os diagnósticos de angiodisplasia e de neoplasia do trato digestivos não são rotineiramente feitos. O questionamento do artigo é válido por vários aspectos: reforça a importância de pesquisar ativamente a presença de angiodisplasia nessa população e, com isso, justifica um novo olhar sobre as indicações habituais de intervenção na estenose aórtica. Outro ponto de ponderação é o uso criterioso da antiagregação plaquetária nessa população. Outro aspecto interessante levantado nesse estudo é a indicação de intervenção valvar nesses pacientes, independentemente da presença de sintomas ou complicadores, mas, isso ainda é controverso. Por último, diante da singular fragilidade clínica de um paciente com estenose aórtica grave, é sempre importante o seu encaminhamento para centros de referências que se tenham acesso à multiespecialidade e à tomada de decisão mais assertiva.
