# Supplementary material for: Os Pacientes com Síndrome de Heyde Deveriam Sofrer Intervenção Valvar Precoce?
Source: Arq Bras Cardiol. 2021 Sep 1;117(3):518–9. [Article in Portuguese] doi: 10.36660/abc.20210734 (PMC8462950; doi:10.36660/abc.20210734)
Supplement: Translation

# Translation: Should Patients with Heyde’s Syndrome Undergo Early Valve Intervention?

**Affiliations:** 1 João Pessoa PB Brazil Universidade Federal da Paraíba, João Pessoa, PB – Brazil; 2 Belo Horizonte MG Brazil Faculdade Ciências Médicas de Minas Gerais, Belo Horizonte, MG – Brazil; 3 São Paulo Brazil Instituto do Coração (InCor) do Hospital das Clínicas da Faculdade de Medicina da Universidade de São Paulo, São Paulo – Brazil

**Keywords:** Aortic Valve Stenosis, Gastrointestinal Hemorrhage/complications, Percutaneous Coronary Intervention, Comorbidities, Mortality

The epidemiological profile of patients with aortic stenosis has changed with the aging of the population, characterized by an increase in associated comorbidities.^1^ Thanks to the revolution in percutaneous aortic valve treatment, many patients who previously would have limited survival and quality of life have benefited from the possibility of curative treatment. On the other hand, the clinical challenges that arise in this population reinforce not only the consolidated relevance of the heart team but also of other specialties such as oncology and hematology.

The interesting study published in the current issue reinforces the importance of the association between Heyde’s syndrome and aortic stenosis, with increased bleeding in this population. However, it is important to highlight in this context the frequently associated comorbidities that may contribute to eventual digestive bleeding, such as the association between aortic stenosis and neoplasm, since both share the same risk factors, particularly colon cancer neoplasm.^2^ This one presents with cardinal clinical signs: anemia and low gastrointestinal bleeding. In this context, the exclusion of neoplasm is mandatory, even in the suspicion of Heyde’s Syndrome. We can also describe other frequently associated comorbidities^3^ that add bleeding risk due to the therapy used, such as coronary artery disease, which usually uses antiplatelet therapy, or dual anti-aggregation after percutaneous coronary intervention with a drug-eluting stent.

Another example is the increased prevalence of atrial fibrillation.^4^ In this scenario, when Heyde’s Syndrome is present, there is an intrinsic maximization of bleeding once anticoagulation is used. Another clinical condition that is growing in the elderly population is cardiac amyloidosis, mainly by transthyretin. Another clinical condition growing in the elderly population is cardiac amyloidosis, mainly caused by transthyretin. The association with severe aortic stenosis is more common than previously thought, ranging between 12 and 16%.^5^^,^^6^ Like aortic stenosis, its prevalence is higher in the elderly population; amyloid fiber acts directly in the coagulation cascade, favoring the emergence of spontaneous hemorrhagic events.^7^ Finally, not related to increased bleeding, but the impact that this may cause, the frailty of the patient with aortic stenosis.^8^ A patient with severe aortic stenosis, considered frail, has a less organic reserve to withstand any severe bleeding. It is known that the need for blood transfusion negatively impacts the morbidity and mortality of this population.^9^

The work of Rosa et al.^10^ reinforces the importance of a neglected association with a high rate of severe bleeding and the need for transfusion on admission, with an increased risk of mortality. The current recommendation for screening colonoscopy for colorectal cancer has been reduced to 45 years according to the U.S. Preventive Services Task Force. In addition, access to endoscopic procedures is not massively available to a large part of the population in our country, which increases risk since the diagnoses of angiodysplasia and neoplasia of the digestive tract are not routinely made. The article’s questioning is valid for several aspects: it reinforces the importance of actively researching angiodysplasia in this population. Therefore, it justifies a new look at the usual indications for intervention in aortic stenosis. Another point of weighting is the judicious use of platelet anti-aggregation in this population. Another interesting aspect raised in this study is the indication of valve intervention in these patients, regardless of the presence of symptoms or complicating factors, but this is still controversial. Finally, given the unique clinical fragility of a patient with severe aortic stenosis, it is always important to refer them to reference centers with access to multispecialty and more assertive decision-making.
